# Physico-Chemical Properties of MgGa Mixed Oxides and Reconstructed Layered Double Hydroxides and Their Performance in Aldol Condensation of Furfural and Acetone

**DOI:** 10.3389/fchem.2018.00176

**Published:** 2018-05-24

**Authors:** Oleg Kikhtyanin, Libor Čapek, Zdeněk Tišler, Romana Velvarská, Adriana Panasewicz, Petra Diblíková, David Kubička

**Affiliations:** ^1^Unipetrol Centre for Research and Education Litvínov, Czechia; ^2^Technopark Kralupy VŠCHT Praha, University of Chemistry and Technology Prague Kralupy nad Vltavou, Czechia; ^3^Department of Physical Chemistry, Faculty of Chemical Technology, University of Pardubice Pardubice, Czechia; ^4^Department of Organic Technology, University of Chemistry and Technology Prague Prague, Czechia

**Keywords:** MgGa layered double hydroxides, mixed oxides, reconstructed hydrotalcites, aldol condensation, acido-basic properties

## Abstract

MgGa layered double hydroxides (Mg/Ga = 2–4) were synthesized and used for the preparation of MgGa mixed oxides and reconstructed hydrotalcites. The properties of the prepared materials were examined by physico-chemical methods (XRD, TGA, NH_3_-TPD, CO_2_-TPD, SEM, and DRIFT) and tested in aldol condensation of furfural and acetone. The as-prepared phase-pure MgGa samples possessed hydrotalcite structure, and their calcination resulted in mixed oxides with MgO structure with a small admixture phase characterized by a reflection at 2θ ≈ 36.0°. The interaction of MgGa mixed oxides with pure water resulted in reconstruction of the HTC structure already after 15 s of the rehydration with maximum crystallinity achieved after 60 s. TGA-MS experiments proved a substantial decrease in carbonates in all rehydrated samples compared with their as-prepared counterparts. This allowed suggesting presence of interlayer hydroxyls in the samples. Acido-basic properties of MgGa mixed oxides determined by TPD technique did not correlate with Mg/Ga ratio which was explained by the specific distribution of Ga atoms on the external surface of the samples. CO_2_-TPD method was also used to evaluate the basic properties of the reconstructed MgGa samples. In these experiments, an intensive peak at T = 450°C on CO_2_-TPD curve was attributed to the decomposition of carbonates newly formed by CO_2_ interaction with interlayer carbonates rather than to CO_2_ desorption from basic sites. Accordingly, CO_2_-TPD method quantitatively characterized the interlayer hydroxyls only indirectly. Furfural conversion on reconstructed MgGa materials was much larger compared with MgGa mixed oxides confirming that Brønsted basic sites in MgGa catalysts, like MgAl catalysts, were active in the reaction. Mg/Ga ratio in mixed oxides influenced product selectivity which was explained by the difference in textural properties of the samples. In contrast, Mg/Ga ratio in reconstructed catalysts had practically no effect on the composition of reaction products suggesting that the basic sites in these catalysts acted similarly in aldol condensation of acetone with furfural. It was concluded that the properties of MgGa samples resembled in a great extent those of MgAl hydrotalcite-based materials and demonstrated their potential as catalysts for base-catalyzed reactions.

## Introduction

A common feature of the Layered Double Hydroxides (LDH) or Hydrotalcite-like (HTC) family, both natural and synthesized, is that they all have a structure closely related to that of the mineral hydrotalcite, that is, rhombohedral Mg_6_Al_2_(OH)_16_CO_3_·4H_2_O. All these materials are composed of two-dimensional layers of positively charged double hydroxides together with water molecules and exchangeable charge-compensating anions which located in interlayer (Cavani et al., 1991; Sels et al., 2001; Debecker et al., 2009; Takehira, 2017). The general formula of LDHs can be described as [${\text{M}}_{1-\text{x}}^{2+}$M^3+^_x_(OH)_2_]^x+^ [A_x/n_]^n−^·mH_2_O, where M^2+^ is a divalent cation, M^3+^ is a trivalent cation and A is a charge-compensating anion. Mg-Al hydrotalcites, most known and well-studied among the total family, are derived from brucite Mg(OH)_2_ as a general crystallographic structure. These brucite layers are stacked on top of each other and held together by weak interactions through hydrogen atoms (Cavani et al., 1991; Debecker et al., 2009). In the brucite layers a part of Mg^2+^ cations are substituted with Al^3+^ cations thus creating a positive charge in the layers. In synthetic MgAl HTCs, the substitution degree of Mg → Al may be different but lies in the range of x = 0.1–0.5 (Cavani et al., 1991). A charge resulting from this substitution is compensated by interlayer anions (${\text{CO}}_{3}^{2-}$, ${\text{NO}}_{3}^{-}$, Cl^−^, etc). Additionally, water molecules are in the interlayer in amounts dependent on the temperature, on the water vapor pressure and the nature of the anions present (Cavani et al., 1991; Debecker et al., 2009). The chemical composition of LDHs is not limited to Mg and Al cations, and at the present the family of these compounds consists of a large variety of synthetic materials which are composed of Mg^2+^, Zn^2+^, Co^2+^, Cu^2+^, etc., as divalent cations, and Al^3+^, Fe^3+^, Cr^3+^, La^3+^, etc., as trivalent cations (Cavani et al., 1991; Choudary et al., 2001; Sels et al., 2001; Motokura et al., 2006; Pérez-Ramírez et al., 2007a; Debecker et al., 2009; Takehira, 2017).

The most popular method of synthesizing LDHs is based on the co-precipitation of aqueous solutions of the corresponding salts (usually nitrates) with alkaline solutions (Na or K hydroxide and carbonate) at low supersaturating conditions and fixed pH values. As a consequence, carbonate groups are present as charge-compensation anions in as-prepared LDHs. The as-prepared materials exhibit low activity in catalytic applications and therefore have to be activated. Heat treatment is the main and the simple way to activate the as-prepared LDHs which results in the removal of water, the dehydroxylation of brucite-like layers and the decomposition of interlayer carbonates. Mixed oxides formed by the thermal decomposition of as-prepared LDHs exhibit much better basic properties than the starting as-prepared LDHs. The mixed oxides possess Lewis basic sites and are widely used in base-catalyzed reactions such as transesterification (Zeng et al., 2008), condensations (Kustrowski et al., 2006; Perez et al., 2009), alkylation (Cavani et al., 2005), and Michael addition (Prescott et al., 2005).

A distinctive feature of HTC-like materials is so-called “memory effect” described for the first time by Miyata (1980), i.e., the recovery of original lamellar structure by hydration of mixed oxide. Thus, the interaction of MgAl mixed oxide either with water vapor or by immersion in decarbonated water leads to the formation of meixnerite [Mg_6_Al_2_(OH)_18_·4H_2_O] which is a hydrotalcite analog with OH^−^ groups as compensating anions in the interlayer instead of the original carbonates (Climent et al., 2002b; Tichit and Coq, 2003; Abelló et al., 2005; Pérez-Ramírez et al., 2007b; Kikhtyanin et al., 2017b). The interlayer hydroxyls are Brønsted basic sites and, therefore, the reconstructed materials are widely used in a number of base-catalyzed-reactions which require Brønsted basicity, such as self- and cross-aldol condensation of aldehydes and ketones (Tichit et al., 1998, 2002; Climent et al., 2002a; Abelló et al., 2005), Michael additions (Choudary et al., 1999), Knoevenagel and Claisen-Schmidt condensation (Cavani et al., 1991; Climent et al., 1995; Guida et al., 1997; Di Cosimo et al., 1998), etc.

In a zeolite family, the substitution of aluminum atoms by gallium in a silicate matrix leads to the formation of gallium silicates of various structural types whose specific physicochemical properties are successfully used in a number of acid-catalyzed reactions (Fricke et al., 2000; Chao and Liu, 2005; Wu et al., 2010). It is therefore not surprising that a possibility to replace Al atoms by Ga atoms is also assumed in other classes of inorganic compounds. Indeed, the synthesis and the study of the physico-chemical properties of MgGa LDHs have been reported repeatedly (Rebours et al., 1994; López-Salinas et al., 1996, 1997; Aramendía et al., 1999a,b, 2000; Thomas and Vishnu Kamath, 2005; Grand et al., 2010). Similar to other LDHs, the synthesis of MgGa is performed starting from Mg and Ga salt solutions mixed with sodium hydroxide and carbonate solutions; the heat treatment of the as-prepared materials results in MgGa mixed oxides. Nevertheless, in most cases, the studies on the properties of the prepared MgGa LDHs and mixed oxides are limited to their synthesis and characterization by different physico-chemical methods, such as XRD, TGA, DRIFT, MAS, NMR. More rarely, studies on the basic properties of MgGa mixed oxides have also been documented (López-Salinas et al., 1997; Prinetto et al., 2000). In addition, the “memory effect” has been demonstrated for this type of materials. Indeed, MgGa mixed oxides restored LDH structure (i) by exposure to a water-saturated atmosphere for 18 h followed by CO_2_ picked up from the ambient for this reconstruction (Thomas and Vishnu Kamath, 2005), (ii) by the dispersion of MgGa mixed oxide under vigorous magnetic stirring during 1 h into the decarbonated water at 298 K and 1 × 10^5^ Pa (Prinetto et al., 2000), or (iii) by the treatment of MgGa mixed oxide with a carbonate-containing aqueous solution (López-Salinas et al., 1996). Despite the growing interest to Ga-containing catalysts in different applications, there is a lack of available information which reports about their catalytic performance in base-catalyzed reactions. Concerning Ga-containing MgGa LDHs, Prinetto et al. (2000) demonstrated that the substitution of Al^3+^ with Ga^3+^ slightly increased the density of the basic sites in the Mg-containing mixed oxides and slightly increased their catalytic activity in acetone self-condensation. Tabanellia et al. (2018) used MgGa mixed oxide for the gas-phase methylation of phenol to 2,4,6-trimethylphenol and attributed the outstanding performance of the catalyst to its high activity in methanol dehydrogenation to formaldehyde as well as to the moderate acidic features due to Ga sites, which enhanced the intramolecular rearrangement of O-alkylated compounds. Rousselot et al. (1999) investigated the catalytic performance of both as-prepared and calcined MgGa LDHs in Knoevenagel condensation of benzaldehyde with ethyl cyanoacetate. They explained the obtained results by a rehydration process of the calcined samples during the catalytic reaction.

Nevertheless, there is a great lack in information about the performance of catalysts based on reconstructed MgGa LDHs. Moreover, in contrast to MgAl materials (Pérez-Ramírez et al., 2007b; Kikhtyanin et al., 2017b), the effect of reconstruction time on the physico-chemical properties of MgGa LDHs and the catalytic performance of the reconstructed materials has not been reported yet.

**Scheme 1:**
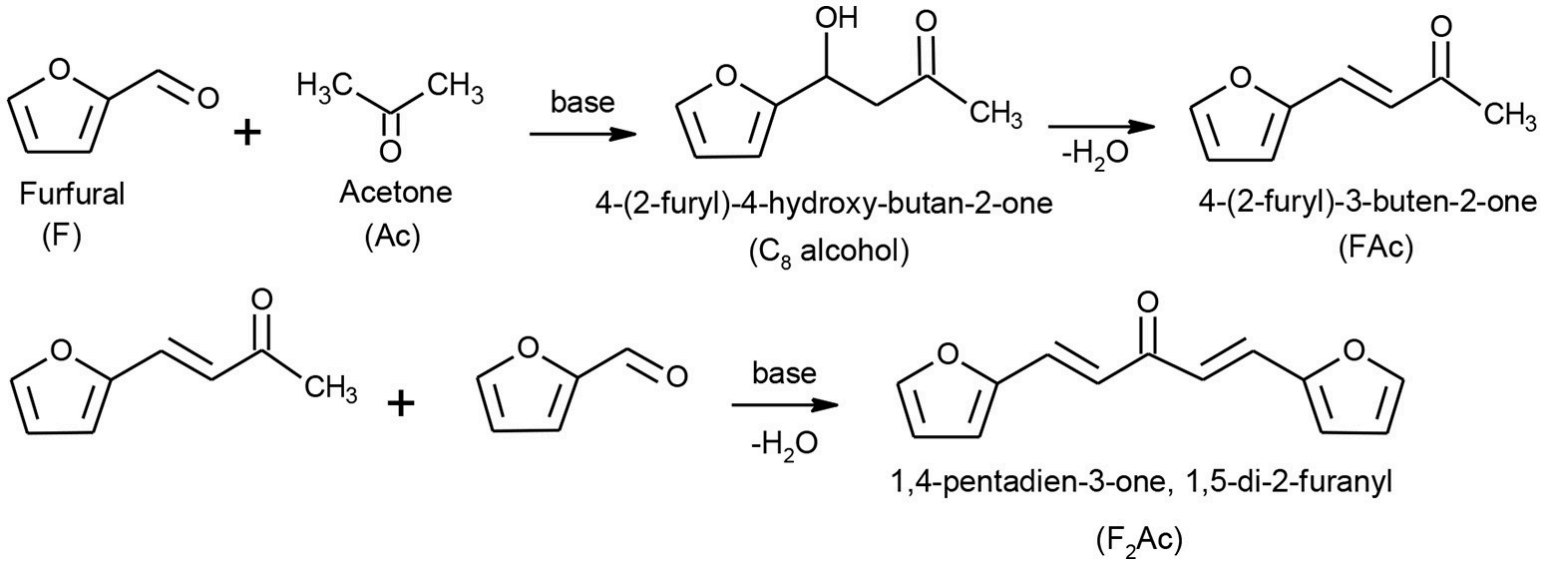
Reaction scheme of aldol condensation between furfural and acetone.

Aldol condensation of furfural and acetone (Scheme 1) is an attractive object for an investigation from several points of view. First of all, this reaction has a great practical potential as it allows increasing the carbon atom chain length starting from the relatively simple ones which can be produced by biomass processing (Gámez et al., 2006; Mäki-Arvela et al., 2011). The obtained condensation products can be further hydrogenated/deoxygenated to afford hydro-carbons, namely C_8_ and C_13_ alkanes (Zapata et al., 2012; Ramos et al., 2016). On the other hand, aldol condensation of furfural and acetone attracts also a scientific interest, because this reaction makes it possible to probe both the acid (Kikhtyanin et al., 2014, 2015) and basic sites (Sádaba et al., 2011; Faba et al., 2012; Thanh et al., 2016) of heterogeneous catalysts. Regularities found in preceding studies help to evaluate and understand the catalytic performance of following catalytic materials.

The purpose of this paper is to correlate the physicochemical characteristics of samples derived from MgGa LDHs varied by Mg/Ga ratio with their catalytic performance. A special attention is paid to the reconstructed MgGa materials, namely, a possibility to recover HTC structure by the interaction of MgGa mixed oxide with pure water. For this purpose, the effect of rehydration duration of MgGa mixed oxides on the properties of the obtained samples was studied in detail and the catalytic performance of both MgGa mixed oxides and reconstructed LDHs were compared in aldol condensation of furfural and acetone.

## Materials and methods

### Preparation of MgGa mixed oxides

MgGa layered double hydroxides varied in Mg:Ga molar ratio in reactive mixture in the range of (2–4):1 were prepared based on a method described in Hora et al. (2015). For these syntheses Ga nitrate was prepared by the dissolution of powder metallic Ga (Unimagnet) in concentrated nitric acid followed by the evaporation of the excessive acid by using vacuum evaporator. The composition of obtained salt was determined by ICP. MgGa LDH's with Mg:Ga molar ratio from 2:1 to 4:1 were synthesized by co-precipitation method at constant pH value (pH = 9.5) and constant temperature (T = 60°C). The preparation procedure involves mixing of aqueous solutions of nitrates consisting of Gallium nitrate Ga(NO_3_)_2_·6H_2_O prepared as described above and magnesium nitrate Mg(NO_3_)_2_·6H_2_O (Lach-ner, p.a. purity) (c_Mg+Ga_ = 1 mol/dm^3^), and a basic solution containing potassium carbonate K_2_CO_3_ (Penta, p.a. purity) and potassium hydroxide KOH (Lach-ner, p.a. purity) (c_KOH_ = 2 mol/dm^3^ + c_k_2_*co*_3__ = 0.2 mol/dm^3^). After precipitation the solids were isolated by press-filtration using paper filter plate S15N (Hobra); the filter cake was washed by demineralized water to neutral pH and dried in oven overnight at 65°C.

The MgGa mixed oxides were prepared by calcination of the dried as-prepared LDHs at 450°C for 3 h (heating rate 5°C·min^−1^). The rehydration of the mixed oxides with deionized water was performed at room temperature and rehydrate on time in the range of 0–40 min. All samples chosen for physico-chemical characterization and catalytic runs were dried for 40 min. Other details in the preparation of the rehydrated samples are available in Kikhtyanin et al. (2017b). After preparation (rehydration+drying steps), all materials were kept in a desiccator under inert atmosphere to prevent contact of the samples with CO_2_ from air during their storage. The samples were taken away from the desiccator only before performing experiments on their characterization. Further in the text, the as-prepared MgGa LDHs are denoted as MgGa-xA, calcined MgGa mixed oxides are denoted as MgGa-xC and rehydrated samples are denoted as MgGa-xR-y, where x stands for Mg/Ga molar ratio and y stands for rehydration time.

### Physico-chemical characterization

Chemical composition was determined by ICP-OES. The phase composition of the prepared samples was determined by X-ray powder diffraction using a Philips MPD 1880 instrument with Cu K_α_ irradiation (λ = 0.154 nm) in the 2θ range of 5–70° at the 2θ scanning rate of 2.4°·min^−1^. In each group of prepared catalysts, i.e., as-prepared LDHs, MgGa mixed oxides and reconstructed LDHs, the sample with the highest crystallinity was assigned relative crystallinity 100%. Textural properties were determined from N_2_ physisorption isotherms at 77 K obtained by using a Quantachrome AUTOSORB unit. Prior to the analyses, the samples were outgassed at 250°C for 3 h in flowing N_2_. BET equation was used to calculate the specific surface area of the samples. Thermogravimetric analysis (TGA/DTG) of the dried as-prepared LDHs, MgGa mixed oxides and rehydrated samples was performed using a TA Instruments TGA Discovery series equipment operating with a heating ramp of 10°C·min^−1^ from room temperature to 900°C in N_2_ flow. TGA-MS experiments were performed using the same TGA unit equipped with a mass-spectrometer OmniStar GSD 320 (Pfeiffer-Vacuum) with a MID (Multiple Ion Detection) measurement mode, a SEM (Secondary Electron Multiplier) detector, and a quadrupole mass-analyzer. DRIFT spectra were recorded on a Nicolet IS 10 FTIR spectrometer equipped with a DTGS detector and KBr beam splitter. All spectra were collected over the range of 4,000–400 cm^−1^ at a spectral resolution of 4 cm^−1^ and number of scans 128 (both for the background and the sample spectra).

Samples for Scanning Electron Microscopy (SEM) observations were mounted on a holder and sputter-coated (Q150R ES, Quorum Technologies Ltd., United Kingdom) by 10 nm of gold to neutralize charging-effects and to increase an SE yield at final micrographs. Further, the images of coated samples were acquired using field emission scanning electron microscope (Lyra3 GMU, Tescan Orsay Holding a.s., Czech Republic) at an accelerating voltage of 12 kV and absorbed current ranging from 200 to 300 pA. For imaging, the SE detection was used to investigate the morphology of samples. The temperature-programmed desorption of carbon dioxide (CO_2_-TPD) or ammonia (NH_3_-TPD) was used to evaluate basic and acidic properties of MgGa mixed oxides and reconstructed LDHs. The details of the methods are presented in Kikhtyanin et al. (2017a). Maximum temperature in TPD experiments was chosen as 450°C which is temperature used for the preparation of MgGa mixed oxides.

### Catalytic test

Furfural (Sigma-Aldrich) and acetone (LachNer, Czech Republic) used for catalytic experiments were pre-dried with a molecular sieve 3A to exclude the effect of moisture originating from the chemicals.

For catalytic experiments with MgGa mixed oxides, 0.5 g of freshly calcined HTC was used. For catalytic experiments with reconstructed MgGa LDHs, 0.5 g of freshly calcined HT was pre-rehydrated according to the method described above.

Aldol condensation of furfural with acetone was carried out in a 100-ml stirred batch reactor (a glass flask reactor) at temperature of 50°C in the case of mixed oxides or 25°C in the case of reconstructed LDHs. Prior to the catalytic tests, the mixture of 19.7 g of acetone and 6.5 g of furfural (acetone to furfural molar ratio 5/1) was stirred at 200 RPM and kept at the predetermined reaction temperature. After that, a studied catalyst (grain of 0.25–0.5 mm) was added and the reaction was carried out at predetermined temperature for 120 min at 200 RPM. It was previously established that the reaction is limited neither by external nor internal mass transfer under the chosen reaction conditions (in tests with changing stirring rate and catalyst particle size; Hora et al., 2015). Samples of liquid products were periodically withdrawn from the reactor during the experiment, filtered and analyzed by Agilent 7890A GC unit equipped with a flame ionization detector (FID), using a HP-5 capillary column (30 m/0.32 mm ID/0.25 μm). Catalytic results of aldol condensation of furfural and acetone were described by conversion and selectivity parameters that were calculated as follows:

$$\begin{array}{l} \text{reactant conversion }(\text{t})\;(\text{mol}\%)=\;100\;\times ({\text{reactant}}_{\text{t = 0}}\\ \;-{\text{reactant}}_{\text{t}}){\text{/reactant}}_{\text{t = 0}}; \end{array}$$

$$\begin{array}{l} \text{selectivity to product i }=\;(\text{mole of reactant converted to product i})\\ \;/(\text{total moles of reactant converted}). \end{array}$$

Carbon balance was monitored in all experiments as the total number of carbon atoms detected in each organic compound with C_n_ atoms (where *n* = 3, 5, 8, …, etc.) divided by the initial number of carbon atoms in F+Ac feed:

$$\begin{array}{l} \text{C balance }(\%)=\;(3_{\text{molC3}}{+5}_{\text{molC5}}\text{+}\ldots {\text{nmol}}_{\text{Cn}})\\ \;/({\text{3molC}}_{\text{3}(\text{t = 0})}\text{ + }{\text{5molC}}_{\text{5}(\text{t = 0})}). \end{array}$$

## Results and discussion

### Chemical, structural, and textural properties of as-prepared LDHs and mixed oxides

ICP data showed that gallium content in the prepared samples was higher than the values calculated based on the composition of the chemical mixture used for the synthesis. Similar deviation from the theoretical composition was observed for MgGa LDHs repeatedly (López-Salinas et al., 1996; Aramendía et al., 1999a,b) what was explained by the considerable solubility of intermediate Ga(OH)_3_ species in a basic solution (López-Salinas et al., 1996).

**Figure 1:**
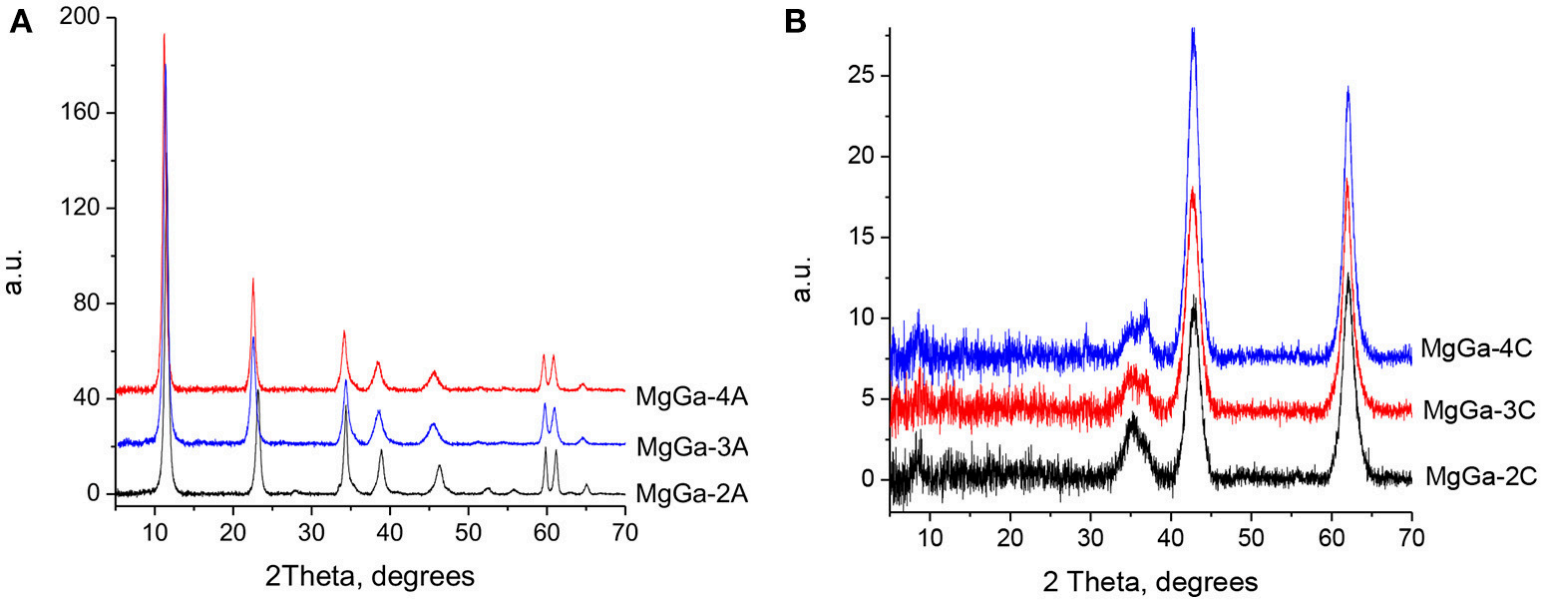
XRD patterns of as-prepared MgGa LDHs **(A)** and mixed oxides **(B)**.

The XRD data confirmed that Ga was highly efficiently incorporated into the brucite-Iike layers of MgGa LDHs in a wide range of Mg/Ga molar ratios. XRD patterns of the as-prepared MgGa LDHs varying by gallium content (Figure 1) show the intensive symmetric lines of a pure hydrotalcite phase (similar to JCPDS Card No. 22-0700). The reflections at 2θ ≈ 11.2°, 22.8°, 36°, and 60° are characteristic for the brucite-like layers (Di Cosimo et al., 1998; Abelló et al., 2005; Kikhtyanin et al., 2017a). The absence of additional lines in the diffractograms suggests that no other crystalline phases are present in the samples thus proving the high phase purity of the as-prepared MgGa LHDs materials. The preparation of phase-pure MgGa LDHs has also been reported in other studies (Rebours et al., 1994; Aramendía et al., 1999b; Thomas and Vishnu Kamath, 2005). MgGa-3A possesses the highest crystallinity; the other two samples show crystallinity of 93–95% relative to MgGa-3A.

The diffraction peaks assigned to (003) and (110) reflections (i.e., at 11.2 and 60°) were used to calculate the basal spacing between the layers (*d*) and unit cell dimension a (as a = 2*d*_110_), respectively. Both the *d* and *a* values increase with the increasing Mg/Ga molar ratio (Table 1), which is an usual trend observed for MgAl hydrotalcites with different Al content (Yun and Pinnavaia, 1995; Di Cosimo et al., 1998; Kikhtyanin et al., 2017a). The increase in the spacing between layers in LDH structure is unequivocally ascribed to differences in the ionic radii of Ga^3+^ and Mg^2+^ being 0.62 and 0.72 Å, respectively^1^, proving the isomorphous substitution of Mg^2+^ by Ga^3+^ atoms within the brucite-like layers. The calcination of the as-prepared materials at T = 450°C results in total destruction of the LDH structure as evidenced by the disappearance of diffraction lines corresponding to the HTC structure. Two intensive diffraction lines observed in XRD patterns of all MgGa mixed oxides at 2θ ≈ 43.0° and 62.5° and a smaller diffraction peak at 2θ ≈ 37.0° are typical for MgO periclase-type structure (JCPDS card No. 45-0946). Similar XRD patterns were observed also after heat treatment of MgAl hydrotalcites (Yun and Pinnavaia, 1995; Di Cosimo et al., 1998; Kikhtyanin et al., 2017a). The relative crystallinity of the MgO phase decreased dramatically with the decline in the Mg/Ga ratio of the mixed oxides (Table 1) suggesting the presence of amorphous phase at high Ga content. The decrease in the MgO basal spacing, d(200), from 2.112 to 2.105 Å with the increasing Ga content (Table 1) indicates that Ga replaced partially Mg in the MgO crystalline framework. Besides, an additional reflection is present in XRD patterns of MgGa mixed oxides at 2θ ≈ 36.0°. It becomes more intensive with the increasing Ga content suggesting that it is originated from a Ga-containing compound. The appearance of an additional line in XRD patterns of MgGa mixed oxides after the calcination of MgGa LDHs at moderate temperatures was reported earlier in several studies (Rebours et al., 1994; Aramendía et al., 1999b; Grand et al., 2010). Rebours et al. (1994) suggested that the reflection at 2θ ≈ 36.0° was either due to the presence of magnesium gallate, MgGa_2_O_4_, or due to the presence of Ga cations in the tetrahedral sites in magnesia lattice.

**Table 1:** Phase composition and BET surface area of the as-prepared hydrotalcites.

**Sample**	**Mg/Ga ratio**	**Phase composi-tion**	**HTC/MgO crystallini-ty, %**	**HTC basal spacing *d*, Å**	**HTC unit cell *a*, Å**	**MgO *d(200)*, Å**	**BET surface, m^2^·g^−1^**
MgGa-2A	2.75	HTC	93	7.69	3.090	–	77
MgGa-3A	4.11	HTC	100	7.78	3.091	–	68
MgGa-4A	5.24	HTC	95	7.90	3.102	–	82
MgGa-2C		MgO	56	–	–	2.105	123
MgGa-3C		MgO	67	–	–	2.111	127
MgGa-4C		MgO	100	–	–	2.112	140
MgGa-2R10		HTC	83	7.86	n.d.	–	5.5
MgGa-3R10		HTC	100	7.88	n.d.	–	5.7
MgGa-4R10		HTC	91	7.96	n.d.	–	3.0

As-prepared MgGa LDHs possess BET surface area in the range of 68–82 m^2^·g^−1^, but the values of BET surface area for MgGa mixed oxides increases to 123–140 m^2^·g^−1^ (Table 1) what is a consequence of the collapse of lamellar HTC structure.

**Figure 2:**
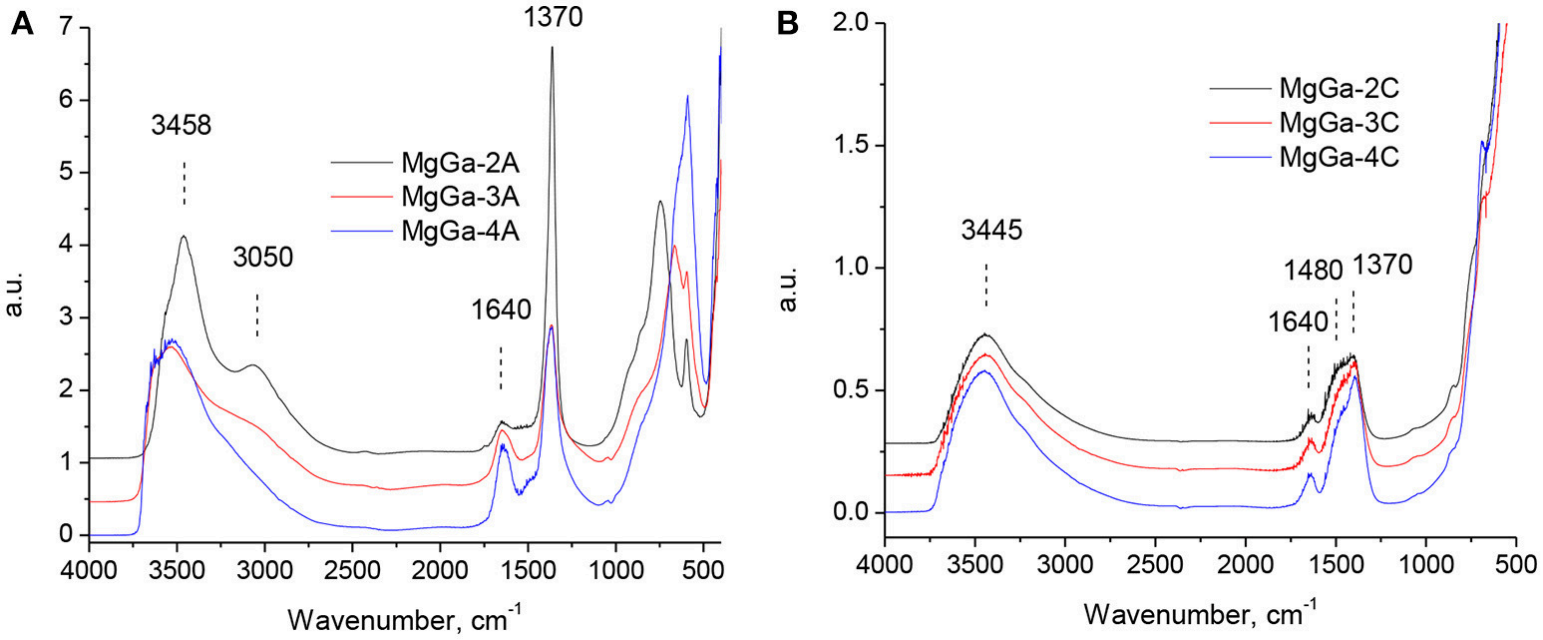
DRIFT spectra of as-prepared MgGa LDHs **(A)** and MgGa mixed oxides **(B)**.

DRIFT spectra of the as-prepared MgGa LDH samples are presented in Figure 2. They agree with those published elsewhere for MgAl (Di Cosimo et al., 1998; Abelló et al., 2005; Kikhtyanin et al., 2017b) and MgGa hydrotalcites (López-Salinas et al., 1996; Aramendía et al., 1999a,b; Thomas and Vishnu Kamath, 2005).

The broad band in the range of 2,700–3,700 cm^−1^ with the maximum at about 3,450–3,550 cm^−1^ is usually attributed to the stretching vibrations of structural hydroxyl groups in the brucite-like layer (Roy et al., 1953) and the twisting vibrations of physisorbed water (Allegra and Ronca, 1978). The shoulder at 3,050 cm^−1^ is assigned to the hydrogen bonding between water molecules and interlayer carbonate anions (López-Salinas et al., 1996). Figure 2 shows that the intensity of this shoulder increases with increase in Ga content. It may be considered as an additional proof that Ga atoms in the composition of brucite-like layers are compensated by interlayer carbonates. The band corresponding to the vibration mode δ_HOH_ at 1,630–1,645 cm^−1^ indicates the presence of interlayer water molecules and the band at 1,370 cm^−1^ arises from the ν_3_ mode of interlayer ${\text{CO}}_{3}^{2-}$ (chelating or bridging bidentate) anions (Abelló et al., 2005). The low intensive band at 1,515 cm^−1^ is ascribed to the reduction in the symmetry caused by the presence of monodentate carbonates (ν _asym__O−C−O_) interacting with Mg^2+^ (Di Cosimo et al., 1998; Abelló et al., 2005). In low frequency region the band at 870 cm^−1^ is characteristic for the out-of-plane deformation of carbonate, whereas the in-plane bending is located at 680 cm^−1^ (Abelló et al., 2005). In Mg-Al hydrotalcites, a band at about 560 cm^−1^ corresponds to the translation modes of hydroxyl groups influenced by Al^3+^ cations (Abelló et al., 2005; Pérez-Ramírez et al., 2007b). Accordingly, the band at 590 cm^−1^ observed in DRIFT spectra of MgGa samples could be attributed to the translation modes of hydroxyl groups influenced by Ga^3+^ cations. Indeed, López-Salinas et al. (1996) proposed that the appearance of this band may be related with Mg-O-Mg or Mg-O-Ga vibrations.

Calcination of the as-prepared MgGa hydrotalcites resulted in a collapse of the lamellar structure accompanied by H_2_O and CO_2_ removal. Correspondingly, DRIFT spectra of the resulting mixed oxides changed significantly (Figure 2B). The intensity of the bands in the range of 2,700–3,700 cm^−1^ substantially decreased due to dehydroxylation. Water removal can be evidenced also by the disappearance of the band at around 1,640 cm^−1^ (water bending vibrations) and of the shoulder at 3,000 cm^−1^ (H_2_O–${\text{CO}}_{3}^{2-}$ interaction in the interlayer). Decomposition of the interlayer carbonates resulted in a decrease in the intensity of the band at 1,370 cm^−1^. A new broad band in the range of 1,400–1,500 cm^−1^ arised from the interaction of non-interlayer carbonates with Mg^2+^ cations on the surface of mixed oxides (Abelló et al., 2005). Additionally, the DRIFT spectra of mixed oxides show that reversible adsorption of water from air can take place during experiments as evidenced by the existence of a small band at around 1,640 cm^−1^ and re-appearance of a broad band with the maximum at 3,450 cm^−1^.

### Thermal treatment of the as-prepared MgGa LDHs to mixed oxides

**Figure 3:**
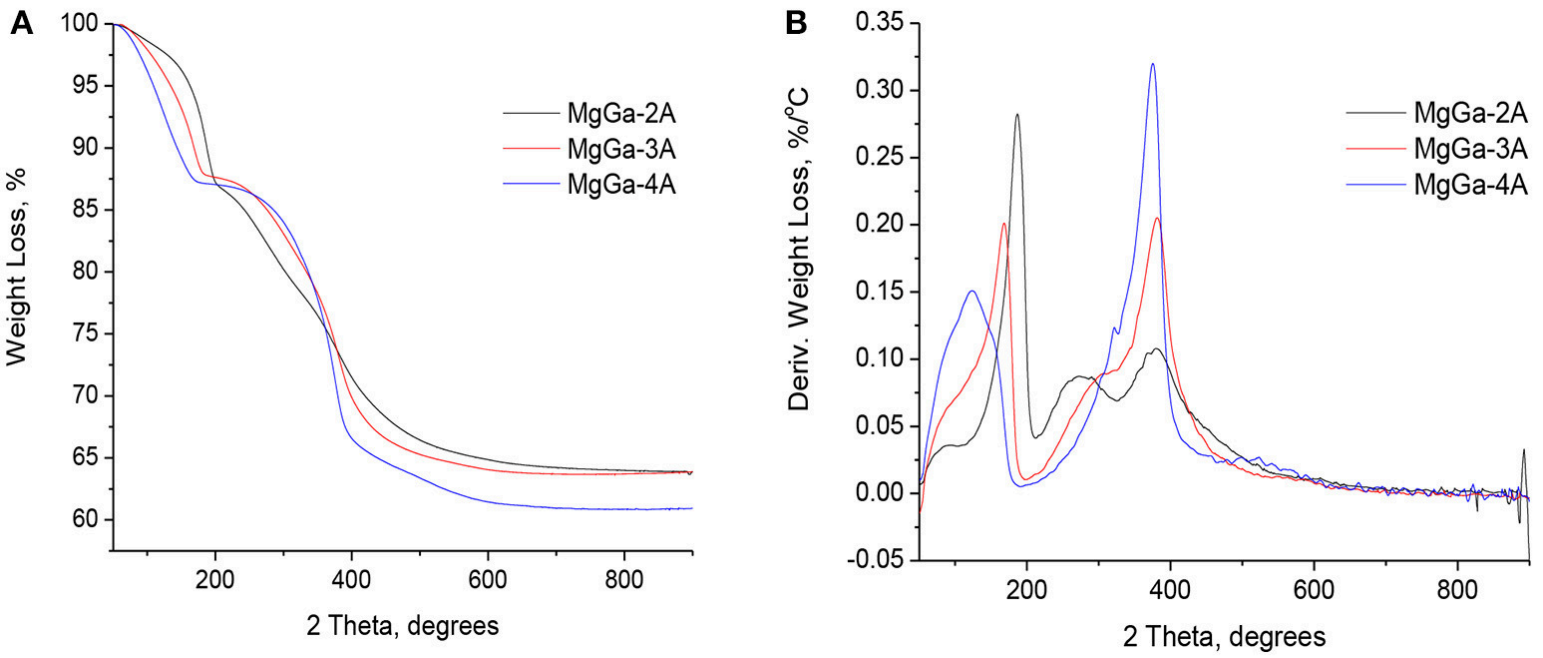
TGA **(A)** and DTG **(B)** curves of as-prepared MgGa hydrotalcites.

Figure 3 depicts the TGA (A) and DTG (B) profiles of the as-prepared MgGa hydrotalcites affording the corresponding MgGa mixed oxides. The total weight loss of the samples is in the range of 36.1–39.1 wt.% (Table 2) and it corresponds well with the data reported earlier for similar materials (López-Salinas et al., 1996; Aramendía et al., 1999a; Thomas and Vishnu Kamath, 2005). The weight loss is 12.2–12.8 wt.% in the temperature range of 50–200°C (Table 2) and it corresponds to the removal of both physically adsorbed and interlayer water molecules. Figure 3B depicts that with increasing Ga content in the as-prepared MgGa LDHs the amount of physisorbed water (T_max._ = 100–120°C) constantly decreases while the amount of interlayer water (T_max._ = 170–185°C) correspondingly increases. The second weight loss of 21.0–22.6 wt.% is observed in the temperature range of 200–500°C and it originates from dehydroxylation of the brucite-like layers and decomposition of carbonates in the interlayer with the corresponding evolution of water and CO_2_, respectively. Figure 3B evidences that the shape of DTG curve in this temperature range depends on Ga content in the samples. MgGa-3A and MgGa-4A have only one predominant signal at T ≈ 380°C, while the DTG curve of MgGa-2A has an additional signal at T = 270°C. Earlier, the presence of more than one kind of OH-groups which differ in properties was suggested in MgAl hydrotalcites with low Mg/Al ratio (Kikhtyanin et al., 2017a). Similarly, the presence of the low-temperature signal in DTG curve of MgGa-2A can be attributed either to the dehydroxylation of defective Ga atoms in the composition of layered structure or to the dehydroxylation of XRD invisible Ga hydroxide phase.

**Table 2:** The results of thermal analysis for the studied MgGa hydrotalcites.

**Sample**	**Weight loss (%)**				**Ratio between signals TGA-MS_H2O_/TGA-MS_CO2_ in the range of T = 200–500^°^C**
	**20–200^°^C**	**200–500^°^C**	**>500^°^C**	**Total**	
MgGa-2A	12.8	21.0	4.3	36.1	4.3
MgGa-3A	12.2	21.3	2.8	36.3	4.8
MgGa-4A	12.8	22.6	3.8	39.2	5.0
MgGa-2R10				53.2	16.4
MgGa-3R10				56.8	21.2
MgGa-4R10				51.3	31.6

Above 500°C, the DTG curve evidences the small additional weight loss of 2.8–4.3 wt.%. Aramendía et al. (1999a) suggested that the final weight loss involves the sustained release of water which results from the residual dehydroxylation of the species with results in Ga_2_O_3_ phase. Nevertheless, without carrying out additional TGA-MS experiments, it is impossible to uniquely assign this signal to a certain species.

**Figure 4:**
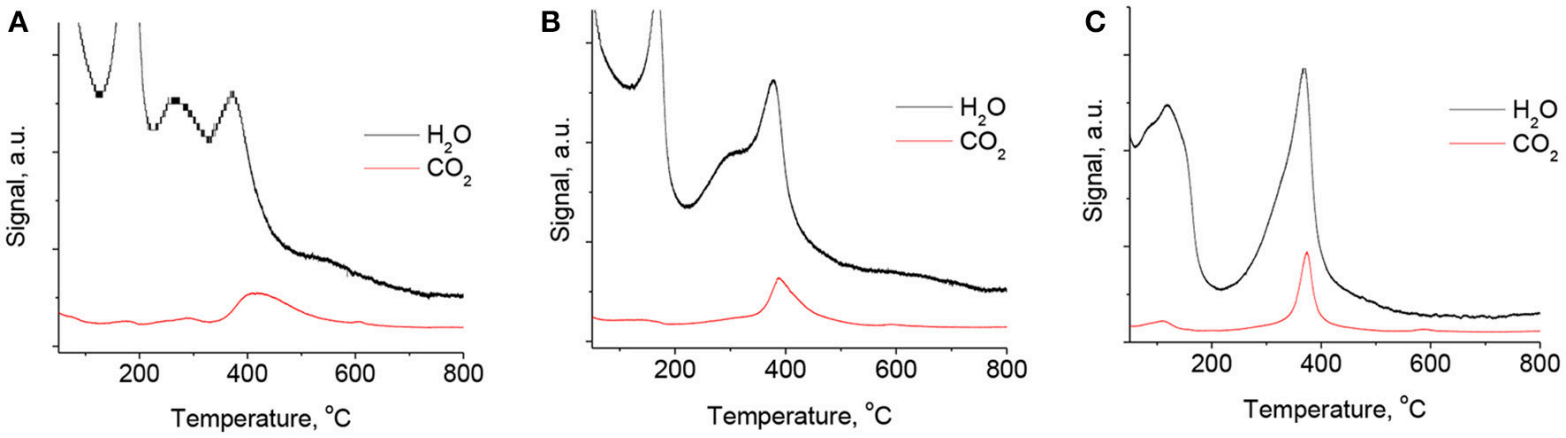
TGA-MS spectra of evolved H_2_O and CO_2_ molecules for MgGa-2A **(A)**, MgGa-3A **(B)**, and MgGa-4A **(C)** samples.

MS curves from the TGA-MS experiments (Figure 4) demonstrate that the dehydroxylation of brucite-like layers and decomposition of charge-compensating carbonates in MgGa hydrotalcites occurs simultaneously in similar temperature range. Nevertheless, the removal of water due to dehydroxylation takes place in a broader range compared to decarbonation (Figure 4) and it ends at temperatures of 550–600°C. It is also seen (Figure 4) that the dehydroxylation of samples with high Ga content occurs in two steps which are characterized by the presence of two peaks at T ≈ 290 and T ≈ 370°C. It allows suggesting that these samples possess two kinds of hydroxyl groups in their composition. Additionally, a slight increase in the TGA-MS-H_2_O profile at temperatures up to 800°C suggests dehydroxylation of the residual OH groups in MgGa mixed oxides, while a small peak at 590°C in TGA-MS-CO_2_ profile can be attributed to the decomposition of residual carbonates. A ratio between areas under TGA-MS-H_2_O and TGA-MS-CO_2_ curves in the temperature range from 200 to 500°C can serve as a measure of the ratio of removed H_2_O and CO_2_ molecules by dehydroxylation and decarbonation, respectively. This ratio increases from 4.3 to 5.0 with an increase in Mg/Ga ratio from 2 to 4 (Table 2) and it reflects the decrease in carbonate groups in the as-prepared samples. It seems to be logical since the theoretical Ga^3+^/${\text{CO}}_{3}^{2-}$ ratio in the as-prepared hydrotalcites is constant and equal to 0.5, while the content of structural hydroxyls is independent on Mg/Ga ratio, so less carbonate groups are present in low-gallium samples.

### Reconstructed MgGa LDH materials

**Figure 5:**
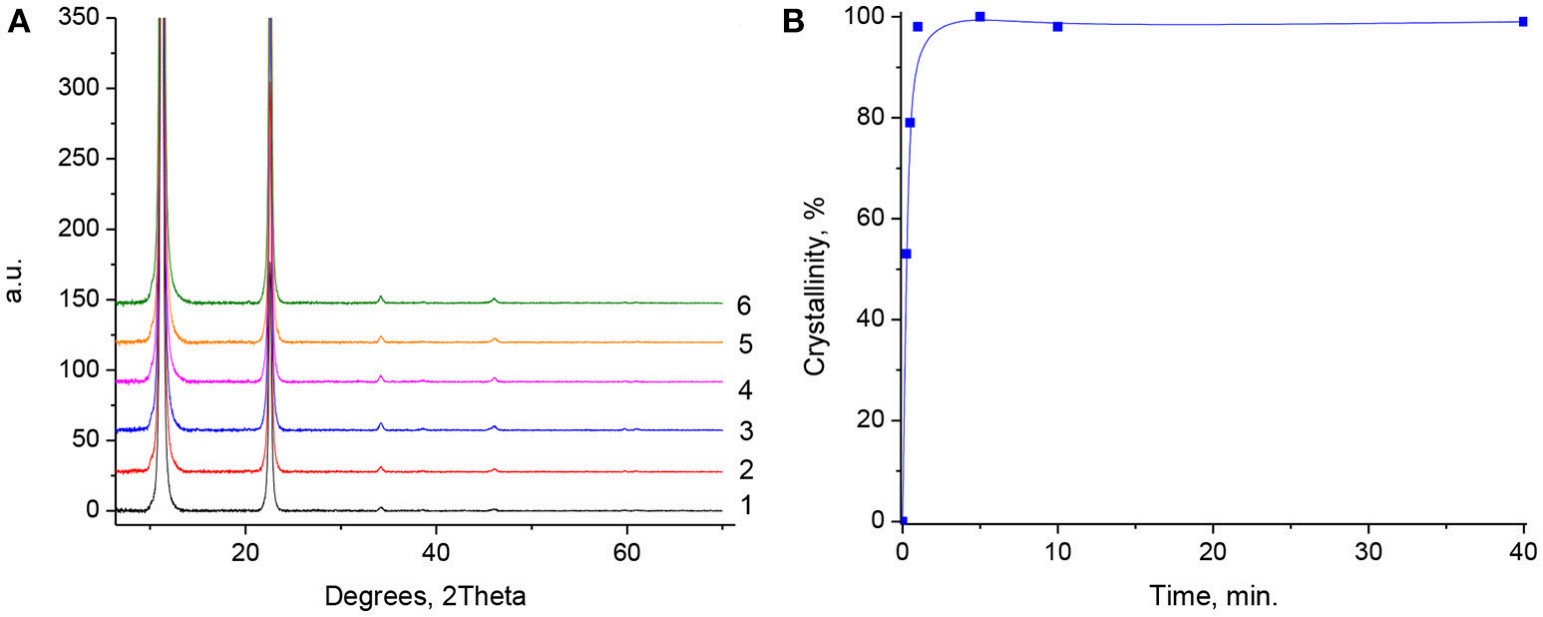
**(A)** XRD patterns of reconstructed MgGa hydrotalcites at different rehydration times. 1-MgGa-3R0.25; 2-MgGa-3R0.5; 3-MgGa-3R1; 4-MgGa-3R5; 5-MgGa-3R-10; 6-MgGa-3R40. **(B)** The dependence of MgGa-3R crystallinity on the duration of MgGa mixed oxides rehydration.

Figure 5A depicts XRD patterns of MgGa-3R0.25 to MgGa-3R40 samples prepared by rehydration of MgGa-3C using decarbonized water under vigorous stirring for different time ranging from 0.25 to 40 min. All prepared samples represent well-crystalline materials with hydrotalcite structure and, therefore, evidence that the prepared MgGa mixed oxides demonstrate a “memory effect” firstly described for MgAl mixed oxides (Miyata, 1980). Figure 5B shows the effect of the duration of the rehydration of MgGa mixed oxide on the crystallinity of the resulting reconstructed LDH. Two main conclusions can be drawn from the observed dependence. Firstly, the crystallinity exceeds 50% already after 0.25 min of rehydration and is close to 100% after 1 min without a visible change with further increase in treatment time. Secondly, Figure 5A shows that the intensity of the XRD reflexes at low 2θ values (< 25°) are higher whereas those at higher 2θ values (>25°) are lower in comparison with the XRD patterns of the as-prepared MgGa LDHs. This difference may be due to a change in the textural characteristics of the reconstructed materials, which will be discussed further when considering SEM results.

The results suggest that the transformation of MgGa mixed oxide occurs very rapidly upon contact with water, similar to the behavior of MgAl mixed oxides (Kikhtyanin et al., 2017b). Only a few minutes of rehydration are enough to get reconstructed MgGa LDHs with maximal crystallinity. In addition, the peak at 2θ ≈ 36°, which was present in the XRD patterns of MgGa mixed oxides (attributed either to the presence of magnesium gallate, MgGa_2_O_4_, or to the presence of Ga cations in tetrahedral sites in the magnesia lattice), is not observed in the XRD patterns of the reconstructed materials. The disappearance of this line designates either the reverse transformation of such a specific MgGa compound into the HTC structure or, at least, these species become XRD-invisible because of a decrease in their size, concentration, or crystallinity during the rehydration treatment.

The basal spacing between the layers (*d*) can be calculated from the position of the peak assigned to (003) reflection, however, the calculation of unit cell dimension (*a*) for the reconstructed samples not possible because of the very low intensity of (110) reflection. Table 1 evidences that the *d*-value calculated for MgGa-2R10, MgGa-3R10, and MgGa-4R10 (Table 1) is less than that for the as-prepared samples. The basal spacing (*d*) can be considered as an indicator for the number of heteroatoms (Ga in this study) in “brucite-like” layers. Therefore, the increase in this value observed for the reconstructed samples allows suggesting that not all Ga atoms are recovered to the crystallographic sites of HTC framework after the rehydration of MgGa mixed oxide. Plausibly they are part of an amorphous phase.

**Figure 6:**
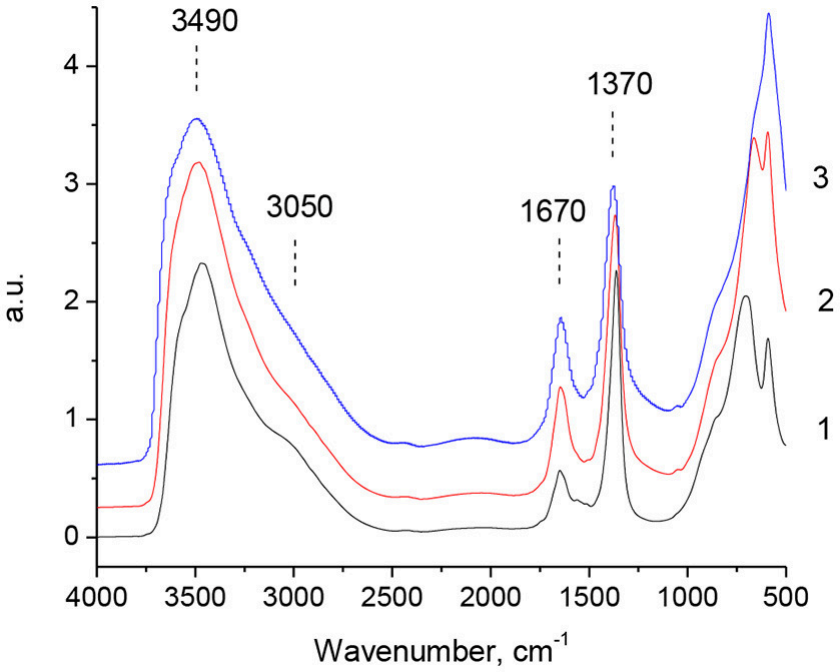
DRIFT spectra for reconstructed MgGa-2R10 (1), MgGa-3R10 (2), and MgGa-4R10 (3).

DRIFT study (Figure 6) provides an additional proof for the existence of the “memory effect” for MgGa samples: DRIFT spectra of the reconstructed materials are very similar to those of the as-prepared materials. Moreover, the spectra do not show any dependence on the rehydration time of MgGa mixed oxides, so Figure 6 depicts only the results for the reconstructed samples prepared by rehydration for 10 min. The recovery of the structural hydroxyl groups in the brucite-like layer is evidenced by an increase in the intensity of the wide band in the range of 2,500–3,600 cm^−1^. The shoulder signal at 3,050 cm^−1^ and the band at 1,670 cm^−1^ re-appear in the spectra, indicating the presence of physisorbed and interlayer water in the prepared samples. The high intensity of the band at 1,370 cm^−1^ suggests the presence of a large number of interlayer compensating anions. As mentioned earlier, this band is attributed to interlayer carbonates in the case of the as-prepared samples. Abelló et al. (2005) noted that this band in the reconstructed MgAl hydrotalcites may also indicate the presence of carbonate groups in the prepared samples due to their contamination with CO_2_ during the rehydration step. However, based on TGA-MS results we proposed that the band at 1,370 cm^−1^ can characterize not only carbonate, but also hydroxyl groups in the interlayer (Kikhtyanin et al., 2017b). Accordingly, we believe that the band at 1,370 cm^−1^ present in the spectra of MgGa reconstructed materials is mostly due to hydroxyl groups rather than carbonate groups. In the analysis, we considered the differences in the signals from H_2_O and CO_2_ in TGA-MS spectra (see below).

According to TGA results, the total weight loss of the reconstructed MgGa samples is in the range of 51–57% (Table 2), which is larger than expected based on the composition of these samples. As shown in Kikhtyanin et al. (2017b), this can be explained by the presence of excessive physisorbed water in the reconstructed samples, which is not removed during the drying of the samples after rehydration. In this case, the TGA method gives only general information about the increase in weight of the obtained samples. More useful information can be obtained by using the TGA-MS method, which allows estimating the relative amount of released H_2_O and CO_2_ molecules and, consequently, the relative content of hydroxyl and carbonate groups in the reconstructed MgGa LDHs.

**Figure 7:**
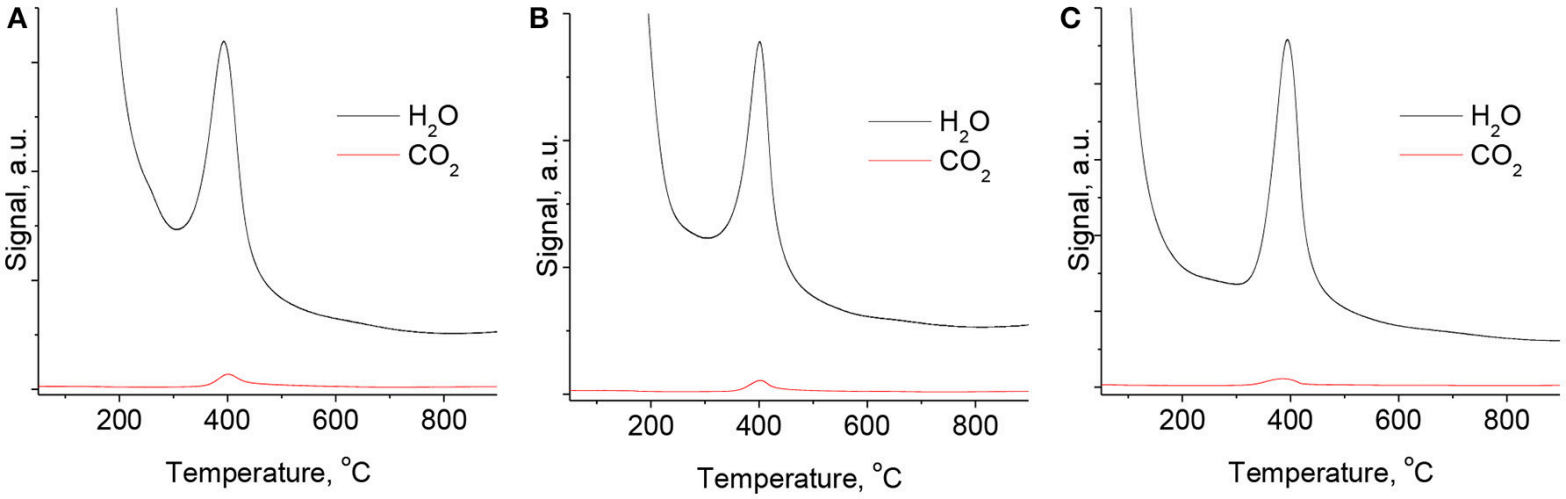
TGA-MS spectra for reconstructed MgGa-2R10 **(A)**, MgGa-3R10 **(B)**, and MgGa-4R10 **(C)**.

There were observed approximately similar intensities of TGA-MS-H_2_O and TGA-MS-CO_2_ signals of reconstructed samples with different Mg/Ga molar ratios. Also, the TGA-MS-H_2_O and TGA-MS-CO_2_ profiles did not show a significant dependence on the rehydration duration. Therefore, Figure 7 depicts the selected characteristic TGA-MS profiles of reconstructed samples with different Mg/Ga ratios and the same rehydration time (10 min) of the corresponding mixed oxides. Figure 7 evidences that the intensity of the signal in TGA-MS-CO_2_ profiles is substantially lower for all the rehydrated samples compared with their as-prepared MgGa LDHs counterparts (Figure 4).

Table 2 shows the range of ratios calculated from the areas under the TGA-MS signals of H_2_O and CO_2_ evolution in the range of 200–500°C. This ratio continuously increases from 16.4 to 31.6 with growing Mg/Ga ratio in the reconstructed MgGa samples. In an ideal case, carbonate groups should be completely absent in the reconstructed MgGa LDHs. In the prepared reconstructed MgGa samples, the presence of carbonates can have two causes. Firstly, MgGa mixed oxides may contain a certain amount of residual carbonates that have not be decomposed during the thermal treatment of the corresponding MgGa LDHs. Indeed, TGA-MS spectra (Figure 4) evidence residual CO_2_ evolution at temperatures above 450°C, which is calcination temperature for the as-prepared materials. Secondly, the reconstructed LDHs can be accidentally exposed to CO_2_ from air during the rehydration/drying processes involved in their preparation and/or during the TGA experiment. In any case, it is practically impossible to avoid completely the presence of carbonates in the reconstructed MgGa materials.

### SEM

**Figure 8:**
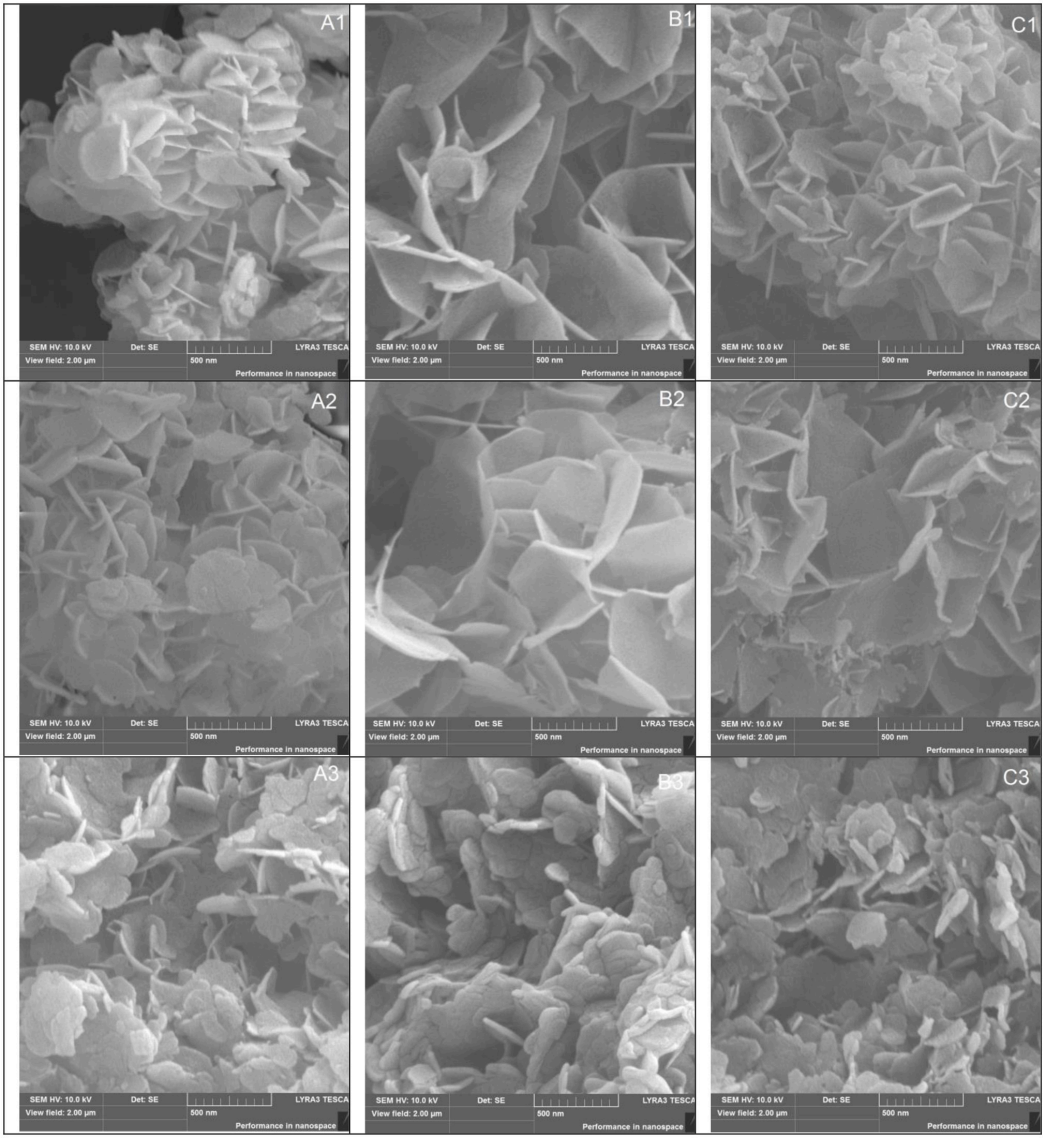
SEM images of MgGa as-prepared materials (A1–C1), mixed oxides (A2–C2), and reconstructed LDHs rehydrated during 10 min (A3–C3). A, Mg/Ga = 2; B, Mg/Ga = 3; C, Mg/Ga = 4.

SEM images were recorded to investigate the morphology of MgGa materials with different Mg/Ga ratio. The micrographs of the as-synthesized LDHs with Mg/Ga molar ratio in the range of 2–4 (Figure 8) show a well-developed layered structure which is typical for of hydrotalcite-like materials.

MgGa-3A with the highest crystallinity was formed by well-developed large platelet aggregates with the size in the range of 5–10 μm consisting of thin, hexagonal, plate-like crystals of 1.5–2.5 μm in length (Figure 8A1). MgGa-2A with higher Ga content was formed by smaller aggregates of ≤ 5 μm in size (Figure 8B1). They were composed of plate-like crystals which were smaller in size (0.7–1.5 μm) and significantly thinner than those in Mg/Ga = 3. This tendency was observed previously also for MgAl LDHs (Kikhtyanin et al., 2017a). MgGa-4A with lower Ga content was built of large massive agglomerates where the plate-like crystals had the size of 1.7–3.5 μm (Figure 8C1). The SEM images of the as-prepared MgGa LDHs also evidence that MgGa-3A has the largest size of individual platelets.

MgGa mixed oxides obtained upon calcination at 450°C maintained a lamellar structure (Figures 8A2–C2). Moreover, the morphology and the size of plate-like crystals and agglomerates were very similar to that of the corresponding as-prepared samples. Nevertheless, the morphology of the crystals of the reconstructed MgGa LDHs was significantly different from the as-prepared materials and mixed oxides. Figures 8A3–C3 depict the SEM images of the reconstructed MgGa materials prepared by rehydration of the corresponding MgGa mixed oxides for 10 min. First of all, separate plate-like crystals became stacked together and created large unshaped agglomerates with the size ≥20 μm in MgGa-3R10 and ≥10 μm in MgGa-2R10 and MgGa-4R10. The size of the individual platelets in the crystals of the reconstructed MgGa LDHs was noticeably smaller, but the thickness of the platelets slightly increased in comparison with the as-prepared materials and mixed oxides. The shape of these platelets became irregular and more defective after rehydration. Finally, Figure 8B3 evidences that the surface of the platelets was cracked. It is obvious that rehydration of mixed oxides had a significant effect on the morphology of the resulting crystals of rehydrated LDHs even though their layered character was preserved. Because of these transformations, the intensity of reflexes in XRD patterns of the reconstructed MgGa LDHs also changed in comparison with the as-prepared materials.

### Acid-base properties

**Figure 9:**
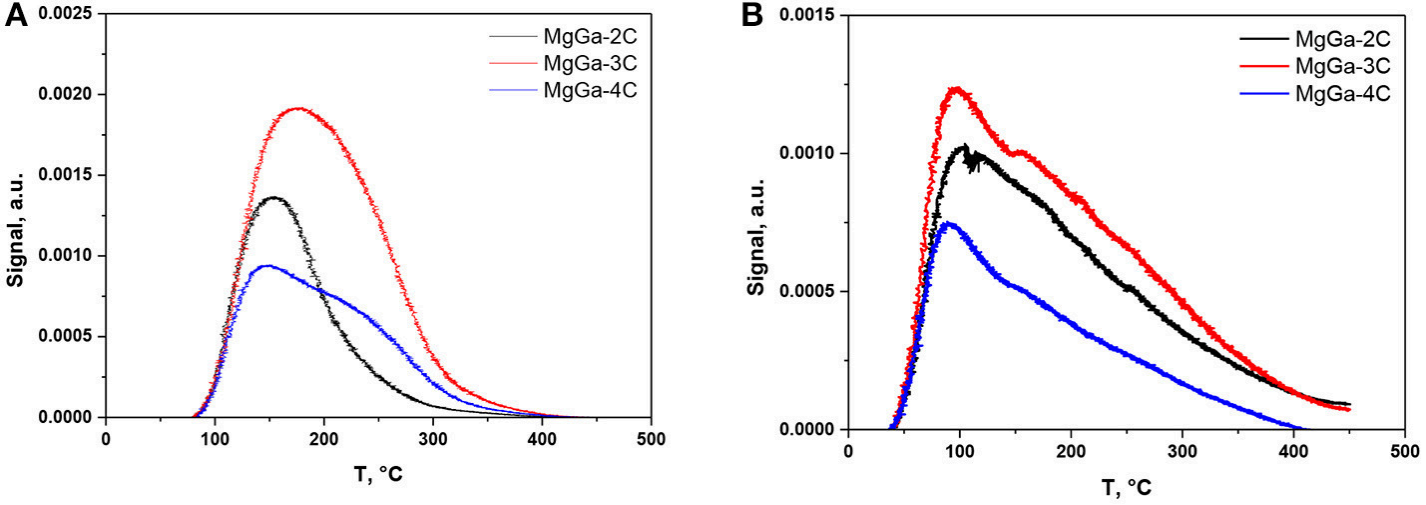
**(A)** NH_3_-TPD and **(B)** CO_2_-TPD profiles of MgGa mixed oxides.

Figure 9A depicts the NH_3_-TPD profiles of MgGa mixed oxides with different Mg/Ga ratio while Table 3 reports the total concentration of acid sites determined from the total amount of desorbed NH_3_ from MgGa mixed oxides.

**Table 3:** Concentration of acid and basic sites in MgGa mixed oxides determined by TPD of adsorbed NH_3_ and CO_2_, correspondingly.

**Sample**	**Amount of desorbed NH_3_, μmol·g^−1^**	**Amount of desorbed CO**_**2**_, μ**mol**·**g**^**−1**^			**Evaluated concentra-tion of interlayer hydroxyls, μmol·g^−1^**	**Theoretical concentra-tion of interlayer hydroxyls, μmol·g^−1^**	**Recovery of Ga atoms by reconstruct-tion process, %**
		**Total**	**L.T**.	**H.T**.			
MgGa-2C	96	149	64	784	1,568	3,784	41
MgGa-3C	190	178	113	770	1,540	3,016	51
MgGa-4C	96	90	88	886	1,772	2,565	69
MgGa-2R10	–	848					
MgGa-3R10	–	883					
MgGa-4R10	–	974					

MgGa-3C possesses the largest concentration of acid sites, 190 μmol·g^−1^, while MgGa-2C and MgGa-4C have similar concentration of acid sites, 96 μmol·g^−1^. A comparison in the shapes of the obtained curves allows suggesting that the mixed oxides contain acid sites varied in their strength. Indeed, two peaks with maximums at about 160 and 250°C can be identified in the TPD profiles (Figure 9A). However, there is no clear dependence of the intensity of individual peaks on Mg/Ga molar ratio. Assuming that the acidity of MgGa mixed oxides should originate from Ga oxidic species, the obtained result is rather curious. Nevertheless, it allows suggesting that the acidity of mixed oxides is not only a consequence of their composition, but other factors, such as presence of admixtures, the distribution of heteroatoms throughout the crystalline framework, etc., should be considered. This implies using broader characterization methods than those used in the present study.

Figure 9B shows the CO_2_-TPD profiles of MgGa mixed oxides. Table 3 gives the total concentration of basic sites derived from the total amount of evolved CO_2_. The TPD curves obtained for all three MgGa mixed oxides have a pronounced maximum between 100 and 120°C. As mentioned previously (López-Salinas et al., 1997; Aramendía et al., 1999a), the peak in the low-temperature region of CO_2_-TPD curve may be assigned to weak basic sites (OH groups). In our previous work, we reported for MgAl mixed oxides that this peak also could reflect the part of medium basic sites (Mg-Al pairs) (Smoláková et al., 2017). Additionally, López-Salinas et al. (1997) observed the second peak on the CO_2_-TPD curve for MgGa mixed oxides, which was present at 200–250°C as a shoulder partially overlapped with the first peak, and a small third peak present as a shoulder above 400°C. The authors ascribed these additional peaks to the appearance of medium and very strong basic sites present in MgGa mixed oxides, respectively (López-Salinas et al., 1997). Taking into account the shape of CO_2_-TPD profiles obtained in the present study, we do not dare to discriminate with certainty between different peaks in the curve and, consequently, we do not provide quantitative contribution of basic sites varied by their strengths to the total basicity of MgGa mixed oxides. Similarly, Aramendía et al. (1999a) reported it was difficult to express the strength of basic sites on an absolute scale and to quantify the number of the sites.

The total concentration of basic sites in the MgGa mixed oxides is 149 and 178 μmol·g^−1^ for MgGa-2C and MgGa-3C, respectively, but it noticeably decreases to 90 μmol·g^−1^ for MgGa-4C. MgGa-3C mixed oxide possesses both the highest amount of acid and basic sites. The dependence of the number of basic sites on Mg/Ga molar ratio follows a general trend between a chemical composition and the total basicity that was earlier observed for MgAl mixed oxides (Kikhtyanin et al., 2017a). Earlier, Di Cosimo et al. (1998) explained the decrease in the basicity observed for MgAl mixed oxides with low Al content by a significant Al surface enrichment. In line with this explanation, it can be assumed that the loss of the total basicity observed for MgGa-4C compared to materials with larger Ga content can also be explained by the specific distribution of Ga atoms on the external surface of the mixed oxides.

CO_2_-TPD is usually used to characterize the basic properties (in terms of both the concentration of basic sites and their distribution by strength) of mixed oxides prepared by heat treatment of LDH materials (López-Salinas et al., 1997; Aramendía et al., 1999a; Di Cosimo et al., 2000; Aramenda et al., 2003; Kikhtyanin et al., 2017a). In contrast, the basic properties of reconstructed hydrotalcites are characterized less often. Abelló et al. (2005) performed an investigation of reconstructed MgAl hydrotalcites by using CO_2_-TPD and identified two peaks in their TPD profile, at around 400–420°C and at ≈550°C. They attributed the observed peaks to two types of basic sites in the rehydrated MgAl mixed oxides. The authors considered the first peak as the contribution of mainly bidentate carbonates, together with bicarbonate species, on the catalyst surface, whereas the second smaller peak was ascribed to monodentate species, similar to those observed in mixed oxides after CO_2_ adsorption.

**Figure 10:**
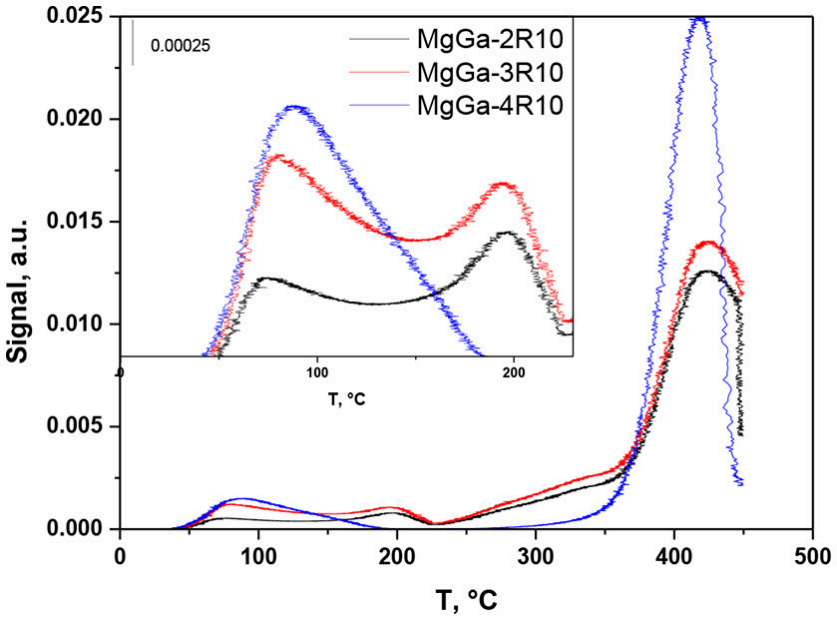
CO_2_-TPD profiles for reconstructed MgGa LDHs.

Figure 10 depicts CO_2_-TPD profiles observed after the interaction of CO_2_ with three reconstructed MgGa materials, MgGa-2R10, MgGa-3R10, and MgGa-4R10. A strong intensive peak with a maximum at around 400°C was observed in the CO_2_-TPD profiles of all the samples. Additionally several smaller peaks can be distinguished between 50 and 230°C. The obtained profiles inevitably indicate the presence of different basic sites in the reconstructed MgGa LDHs.

Table 3 gives the amount of CO_2_ desorbed up to 230°C and between 230 and 450°C. The amount of CO_2_ that desorbed up to 230°C is between 64 and 113 μmol·g^−1^. That amount of CO_2_ is lower compared to the amount of CO_2_ desorbed from the corresponding MgGa mixed oxides (90–178 μmol·g^−1^). However, it has to be mentioned that the absolute amount of CO_2_ desorbed from MgGa mixed oxides and reconstructed MgGa LDHs cannot be directly compared due to the higher amount of water present in the reconstructed materials. The highest amount of desorbed CO_2_ in the range of T ≤ 230°C is observed for the reconstructed MgGa-3R10 material being prepared from mixed oxide MgGa-3C having the highest number of acid and basic sites. Nonetheless, most CO_2_ (between 770 and 886 μmol·g^−1^) desorbed from the reconstructed MgGa materials between 230 and 450°C. This is considerably more than for CO_2_ desorbed from MgGa mixed oxides (90–178 μmol·g^−1^, Table 3).

To explain the origin of the desorption peak between 230 and 450°C in TPD profiles of reconstructed MgGa materials, we did the same experiment as in the case of TPD-CO_2_, but without any adsorption of CO_2_. In that case, only a marginal amount of CO_2_ desorbed from MgGa-3R10 up to 230°C, but 1,186 μmol·g^−1^ of CO_2_ desorbed between 230 and 450°C. The desorbed CO_2_ can be either from an external or an internal source. It is worth noting that water used for rehydration can be excluded as a source of carbonates.

The external origin could be attributed to a dramatic increase in the number of basic sites in the reconstructed MgGa materials compared to MgGa mixed oxides, followed by the rapid interaction of interlayer hydroxyls in freshly prepared reconstructed materials with CO_2_ from air during the preparations for TPD measurements.

The internal origin could be explained by redistribution of CO_2_ from carbonate species that were not decomposed, i.e., that require >450°C to decompose thermally. In a special experiment we checked the amount of residual carbonate species in MgGa-3C mixed oxide and found that the amount of CO_2_ desorbed during a thermal treatment of MgGa-3C from 450 to 900°C is 155 μmol·g^−1^ (not shown). Consequently, the amount of residual carbonates is too low to explain the CO_2_ desorbed from the rehydrated materials between 230 and 450°C. Based on this experiment, the internal origin of carbonates in reconstructed materials can be excluded.

Accordingly, we may conclude that interlayer hydroxyl groups (i.e., charge-compensating anions) in the reconstructed materials readily interact with CO_2_ from air forming interlayer carbonates similar to those present in as-prepared materials. It is evident that the interaction of hydroxyl groups with CO_2_ from air is fast as TPD-CO_2_ experiment with reconstructed MgGa-3R10 material without CO_2_ adsorption followed the rehydration process (the contact of the sample with air could not be excluded). It seems more probable that the intensive peak at around 400°C in the CO_2_-TPD profile of reconstructed MgGa LDHs originates from the decomposition of the newly-formed interlayer carbonates rather than from the decomposition of different species (bidentate or monodentate) on the surface of reconstructed MgGa LDHs, as proposed in Abelló et al. (2005).

If so, the amount of desorbed CO_2_ molecules desorbed between 230 and 450°C in CO_2_-TPD experiments may be considered as a quantitative characteristic of interlayer hydroxyls, which exist in freshly reconstructed LDHs after rehydration treatment. Such assumption can be valid provided that (i) MgGa mixed oxide used for rehydration treatment is substantially free from residual carbonates; and (ii) each CO_2_ molecule during CO_2_-TPD experiments with reconstructed LDHs interacts with two interlayer hydroxyls forming carbonate and water. Based on these assumptions, the concentration of interlayer hydroxyls should be two times larger than the concentration of desorbed CO_2_, i.e., 1,540–1,772 μmol·g^−1^ (Table 3).

The maximum possible concentration of interlayer hydroxyls in reconstructed MgGa LDHs can be calculated from the theoretical composition of the corresponding samples, provided that all Ga are in the crystallographic sites of HTC structure (it is lower as evidenced by XRD data for the reconstructed materials). Table 3 shows that the concentration of interlayer hydroxyls estimated from the amount of desorbed CO_2_ in the range of 230–450°C is lower than the theoretically expected values. Moreover, the increasing Ga content in MgGa LDH should increase the concentration of interlayer hydroxyls. Nevertheless, the obtained results suggest a reverse trend: the evaluated concentration of hydroxyls decreases with the growth of Ga content. It should be however noted that the amount of interlayer hydroxyls in reconstructed materials may be underestimated because not all such hydroxyls in interlayer can be probed by CO_2_, but those located at the edges of the platelets, as proposed by Abelló et al. (2005). Additionally, it should be considered that TPD experiments with the rehydrated materials were terminated at T = 450°C, and this can also contribute to the underestimation of evolved CO_2_, i.e., of interlayer hydroxyls. In any case, the performed CO_2_-TPD experiments give, albeit indirectly, a possibility to evaluate the amount of Brønsted basic sites in reconstructed MgGa materials.

### Catalysis

Before discussing the catalytic results obtained for MgGa catalysts, several related aspects need to be considered. Firstly, on the interaction of reaction mixture with a basic catalyst, both aldol condensation of furfural and acetone self-condensation take place simultaneously. However, in the performed experiments it was found that acetone conversion by self-condensation route did not exceed 2% and therefore it was excluded from further consideration. Secondly, partial dissolution of a catalyst in a reaction mixture may occur under liquid phase conditions which could enable homogeneous reactions. To test this possibility, MgGa-3C was separated from the reaction mixture after 20 min (in a dedicated experiment) and the remaining reaction mixture was stirred for 2 h. The composition of the reaction mixture after 20 min and after the additional 2 h of the experiment was virtually unchanged and catalyst leaching could therefore be excluded. Thirdly, furfural conversion in the presence of the as-prepared MgGa materials was below 0.5% proving that aldol condensation of furfural and acetone required basic sites formed by calcination (MgGa mixed oxides) or calcination followed by rehydration (reconstructed MgGa LDHs) of the as-prepared MgGa LDHs.

**Figure 11:**
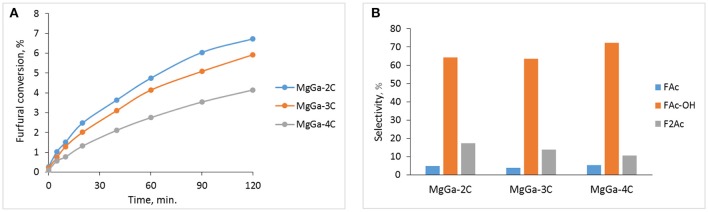
Catalytic properties of MgGa mixed oxides. **(A)** Furfural conversion, **(B)** Selectivity to reaction products at furfural conversion ≈4%. T = 50°C, molar ratio Ac/F = 5.

Figure 11A depicts furfural conversion as a function of reaction time in presence of MgGa mixed oxides with different Mg/Ga ratio at T = 50°C.

Among all studied catalysts, MgGa-2C demonstrated the largest furfural conversion of 6.7% after 120 min of the reaction at T = 50°C (Figure 11A). The increase of Mg/Ga ratio in the MgGa mixed oxides resulted in a consistent decline of furfural conversion. The observed trend in the furfural conversion (Figure 11A) does not show a direct correlation with the concentration of neither acid nor basic sites present in these catalysts (Table 3), and it contrasts with what was usually observed for HTC-derived MgAl mixed oxides (Di Cosimo et al., 1998; Kustrowski et al., 2006; Kikhtyanin et al., 2017a). Therefore, other characteristics of the catalysts should be also considered. For instance, not only the number of active sites, but also their accessibility plays a key role in the observed catalyst activity. Figure 8A2 evidences that the size of the individual platelets of MgGa-2C is lower than that of MgGa-3C. The smaller platelets of MgGa-2C could facilitate the access of reactant molecules to active sites and the removal of reaction products, thus contributing to the increase in furfural conversion. Similarly, Abelló et al. (2005) also discussed the accessibility of active sites in the reconstructed MgAl materials differing in both the size of the platelets and porosity.

Figure 11B shows the product selectivity at furfural conversion ≈4% observed for MgGa mixed oxides with different Mg/Ga ratio. For all catalysts, the selectivity toward FAc is similar (3.9–5.2%), but the selectivity to F_2_Ac obviously increased with the increasing gallium content in the catalysts. In view of the smaller platelets in MgGa-2C, the favorable formation of the second (larger) condensation product seems to be reasonable.

**Figure 12:**
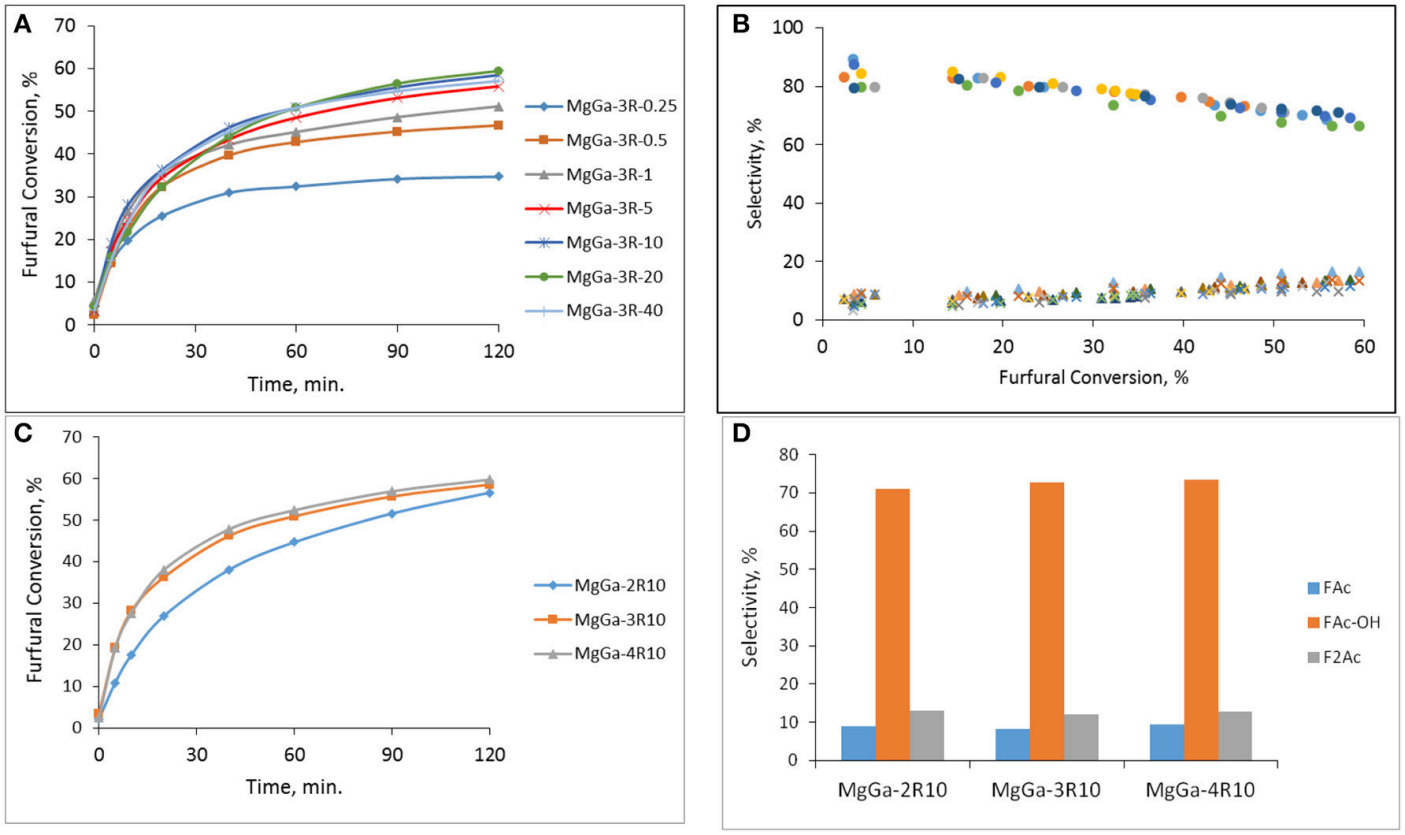
**(A)** The change in furfural conversion in the course of experiment observed on MgGa-3R samples prepared by different rehydration time. **(B)** The dependence of product selectivity on furfural conversion observed on MgGa-3R-X catalysts (∙ – FAc-OH, Δ - FAc, x – F_2_Ac). **(C)** Influence of Mg/Ga in reconstructed MgGaXR-10 samples on furfural conversion. **(D)** Influence of Mg/Ga in reconstructed MgGaXR-10 samples on product selectivity at furfural conversion ≈30%. T = 25°C, molar ratio Ac/F = 5.

Figure 12A demonstrates the dependence of furfural conversion on the duration of catalytic experiment at T = 25°C observed for reconstructed MgGa-3R materials varied by the rehydration time. The furfural conversion is significantly higher compared to the corresponding MgGa mixed oxides (Figure 11A). The observed change in the furfural conversion is in agreement with results obtained earlier for MgAl-derived materials (Kikhtyanin et al., 2017b) thus suggesting that, independently on the chemical composition of LDHs, Brønsted rather Lewis basic sites are favorable for aldol condensation of furfural and acetone. Figure 12A also evidences that the increase in rehydration time resulted in an increased furfural conversion. Such behavior was earlier reported for reconstructed MgAl hydrotalcites (Kikhtyanin et al., 2017b). Taken together, the catalytic performance of both MgAl and MgGa reconstructed LDHs is enabled by rehydration of the corresponding mixed oxides. Regardless of rehydration duration, all reconstructed MgGa LDHs exhibit same product distribution in dependence on furfural conversion (Figure 12B) with selectivity to FAc, FAc-OH, and F_2_Ac being in the range of 8.7–9.8, 76.7–79.2, and 7.8–9.2%, respectively at furfural conversion ≈30%. The similarity in the composition of reaction products regardless the rehydration time allows suggesting that the acid-base characteristics of the catalysts are identical. Consequently, the incomplete reconstruction of HTC framework has no impact on the selectivity but affects the catalytic behavior of the catalysts.

Figure 12C depicts the dependence of furfural conversion on Mg/Ga ratio of reconstructed MgGa materials. The reconstructed MgGa materials with Mg/Ga ratio in the range of 2–4 exhibit the high furfural conversion of 56.6–59.7% after 120 min of the reaction at 25°C. Nevertheless, despite the observed similarity at the end of experiment, the furfural conversion over MgGa-2R10 is lower than that over the other two catalysts, particularly at the beginning of the experiment indicating a lower activity of MgGa-2R10. The observed tendency does not correlate totally with the CO_2_-TPD results for the reconstructed MgGa materials (Figure 10 and Table 3). Indeed, provided that the amount of CO_2_ removed from the samples in CO_2_-TPD experiments in the range of 230–450°C characterizes the amount of interlayer hydroxyls which are Brønsted basic sites, i.e., the active sites of the reaction, the furfural conversion for the reconstructed MgGa LDHs in the reaction should increase in the following order: MgGa-3R10 ≈ MgGa-2R10 < MgGa-4R10. Actually, furfural conversion observed on MgGa-2R10 and MgGa-3R10 differs. As with MgGa mixed oxides, also in the case of reconstructed MgGa LDHs the accessibility of active sites could play a crucial role. In this case a difference in the size of CO_2_ and organic molecules which is responsible for their diffusion to Brønsted basic sites should be taken into account.

A change in Mg/Ga ratio had practically no effect on the composition of reaction products obtained on the reconstructed MgGa LDHs. At furfural conversion of about 30% MgGa-2R10 all reconstructed materials have FAc-OH selectivity of 71–73.5%, FAc selectivity of 8.1–9.3% and F_2_Ac selectivity of 12.1–12.9% (Figure 12D). The obtained results show that, independently on chemical composition, the basic sites in the reconstructed MgGa LDHs act similarly in aldol condensation of acetone with furfural (Scheme 1). Thus, the prepared reconstructed MgGa LDHs exhibit similar trends in catalytic performance in aldol condensation reaction that have been previously observed for MgAl hydrotalcite-derived materials, i.e., the enhanced activity compared to corresponding mixed oxides and the dependence of reaction product composition on the acid-base and textural characteristics of the catalysts. Nevertheless, a direct comparison of the physico-chemical properties and the catalytic performance of MgAl and MgGa mixed oxides and reconstructed LDHs could be considered as the subject of a separate study.

## Conclusion

The results obtained in this paper demonstrate that the synthesis and the characterization approaches developed earlier for Mg-Al LDH-derived materials can be successfully applied in the case of MgGa samples. The heat treatment of as-prepared MgGa LDHs leads to the destruction of HTC structure and the formation of MgGa mixed oxides. These oxidic materials have both acidic and basic sites and they demonstrate an intersiting values of furfural conversion in the aldol condensation of furfural and acetone. The contact of freshly calcined MgGa mixed oxides with pure water results in the fast recovery of HTC structure of MgGa materials, as it is evidenced by XRD, TGA, and DRIFT. The XRD study of the reconstructed MgGa LDHs suggests that after the rehydration process only part of Ga atoms occupy the crystallographic sites of the HTC crystal framework. Nevertheless, the reconstructed MgGa LDHs have significantly higher values of furfural conversion in the aldol condensation of furfural and acetone compared to the corresponding MgGa mixed oxides. Being catalyzed by Brønsted basic sites more effectively, the reaction proves presence of interlayer hydroxyls in the reconstructed MgGa LDHs. Nevertheless, the basic properties of the reconstructed materials cannot be properly characterized by such routine method as CO_2_-TPD because during the experiment CO_2_ as a probe molecule reacts with the interlayer hydroxyls forming interlayer carbonates rather than adsorbed CO_2_ species on basic sites. By the combination of physico-chemical properties and catalytic performance, MgGa mixed oxides and reconstructed MgGa LDHs are analogous to the corresponding Mg-Al counterparts. However, a difference in the nature of the M^3+^ element, Al vs. Ga, which are present in HTC structure should have a significant effect in other applications of these materials, which can be identified in forthcoming studies.

## Author contributions

OK performed and evaluated catalytic experiments and participated in preparing the manuscript. LC evaluated TPD data and participated in preparing the manuscript. ZT prepared MgGa samples and collected the data of physico-chemical characterization. RV carried out and evaluated TGA (TGA-MS) experiments. AP carried out and evaluated TPD experiments. PD carried out and evaluated SEM. DK focused on interpretation of data and participated in preparing manuscript.

### Conflict of interest statement

The authors declare that the research was conducted in the absence of any commercial or financial relationships that could be construed as a potential conflict of interest. The reviewer, IL, and handling Editor declared their shared affiliation.

## Footnotes

^1^*Database of Ionic Radii*. Available online at: http://abulafia.mt.ic.ac.uk/shannon/ptable.php
